# Changing Patterns of Spatial Clustering of Schistosomiasis in Southwest China between 1999–2001 and 2007–2008: Assessing Progress toward Eradication after the World Bank Loan Project

**DOI:** 10.3390/ijerph110100701

**Published:** 2014-01-03

**Authors:** Yi Hu, Chenglong Xiong, Zhijie Zhang, Can Luo, Ted Cohen, Jie Gao, Lijuan Zhang, Qingwu Jiang

**Affiliations:** 1 Department of Epidemiology and Biostatistics, School of Public Health, Fudan University, Shanghai 200032, China; E-Mails: huy@lreis.ac.cn (Y.H.); agao1224@163.com (J.G.); jiangqw@fudan.edu.cn (Q.J.); 2 Key Laboratory of Public Health Safety, Ministry of Education, Shanghai 200032, China; 3 Laboratory for Spatial Analysis and Modeling, School of Public Health, Fudan University, Shanghai 200032, China; 4 Department of Microbiology and health, School of Public Health, Fudan University, Shanghai 200032, China; E-Mail: xiongchenglong@fudan.edu.cn; 5 Department of Environmental Art and Architecture, Changsha Environmental Protection Vocational Technical College, Hunan 410004, China; E-Mail: cherry12204@163.com; 6 Division of Global Health Equity, Brigham and Women’s Hospital & Department of Epidemiology, Harvard School of Public Health, Boston, MA 02115, USA; E-Mail: tcohen@hsph.harvard.edu; 7 National Institute of Parasitic Diseases, Chinese Center for Disease Control and Prevention, Shanghai 200025, China; E-Mail: zhanglijuan727@163.com

**Keywords:** schistosomiasis, spatial pattern, clustering, the World Bank Loan Project (WBLP), hilly and mountainous regions, Southwest China

## Abstract

We compared changes in the spatial clustering of schistosomiasis in Southwest China at the conclusion of and six years following the end of the World Bank Loan Project (WBLP), the control strategy of which was focused on the large-scale use of chemotherapy. Parasitological data were obtained through standardized surveys conducted in 1999–2001 and again in 2007–2008. Two alternate spatial cluster methods were used to identify spatial clusters of cases: Anselin’s Local Moran’s *I* test and Kulldorff’s spatial scan statistic. Substantial reductions in the burden of schistosomiasis were found after the end of the WBLP, but the spatial extent of schistosomiasis was not reduced across the study area. Spatial clusters continued to occur in three regions: Chengdu Plain, Yangtze River Valley, and Lancang River Valley during the two periods, and regularly involved five counties. These findings suggest that despite impressive reductions in burden, the hilly and mountainous regions of Southwest China remain at risk of schistosome re-emergence. Our results help to highlight specific locations where integrated control programs can focus to speed the elimination of schistosomiasis in China.

## 1. Introduction

Schistosomiasis remains a major public health problem in many developing countries in tropical and subtropical regions [1], with more than 200 million people infected and 779 million people at risk [2]. The global burden of schistosomiasis has been estimated at 1.7 to 4.5 million disability-adjusted life years (DALYs) [2], but the true burden is proved to be between four to 30 times greater than previously expected [3]. Of the three main schistosome species, *Schistosoma japonicum* is responsible for human and animal infections in southern China, the Philippines, and parts of Indonesia [4].

China was one of first countries in East Asia to initiate a national schistosomiasis control program. Substantial achievements in fighting against this disease have been made since the mid-1950s [5]. By the end of the 1980s, schistosomiasis was eliminated in four provinces, but the disease remained endemic in eight others [6,7]. In a renewed attempt to eradicate schistosomiasis in China, a 10-year control project was launched in 1992 with a World Bank loan of US$ 71 million and a supplementary fund of US$ 82 million from the Chinese government [8]. This aggressive project employed large-scale chemotherapy, and also included additional interventions such as health education, chemical control of snails and other environmental exposure modifications to reduce exposure. An evaluation of this World Bank Loan Project (WBLP) in 2002 [9] found that infection rates in humans and livestock had decreased by 55% and 50% respectively and that the number of acute infections and of individuals with advanced disease had also significantly decreased. One province that took part in the project, Zhejiang Province (one of the remaining eight endemic provinces), was able to fulfill the national criteria for elimination.

Although clear progress in schistosomiasis control was made during the period of WBLP period, the long-term progress toward elimination has been less clear. For example, in recent years there have been reports of the reemergence of schistosomiasis in some intervention areas [10]. Potential contributors to schistosomiasis reemergence include environmental and socio-political changes, allowing for the establishment of new snail habitats and subsequent rebounds in disease burden [11,12]. Given concern about the durability of the progress against schistosomiasis achieved during the WBLP period, we sought to assess the longer-term progress after the end of this project.

In this study, we investigate temporal changes in the spatial clustering patterns of schistosomiasis in Southwest China to better understand the long-term progress toward eradication after the end of the WBLP. Data from the national schistosomiasis disease registry are used to map the prevalence of schistosomiasis during two periods of time for which these data were available 1999–2001 and 2007–2008. We focus our analyses within the two hilly and mountainous provinces in China in which *S. japonicum* was endemic and where the WBLP interventions were implemented. The first period (1999–2001) allows us to identify spatial disease patterns near the conclusion of the WBLP, while the second period (2007–2008) permits investigation of longer-term changes in disease clustering after the end of the project.

## 2. Materials and Methods

### 2.1. Approach and Study Area

**Figure 1:**
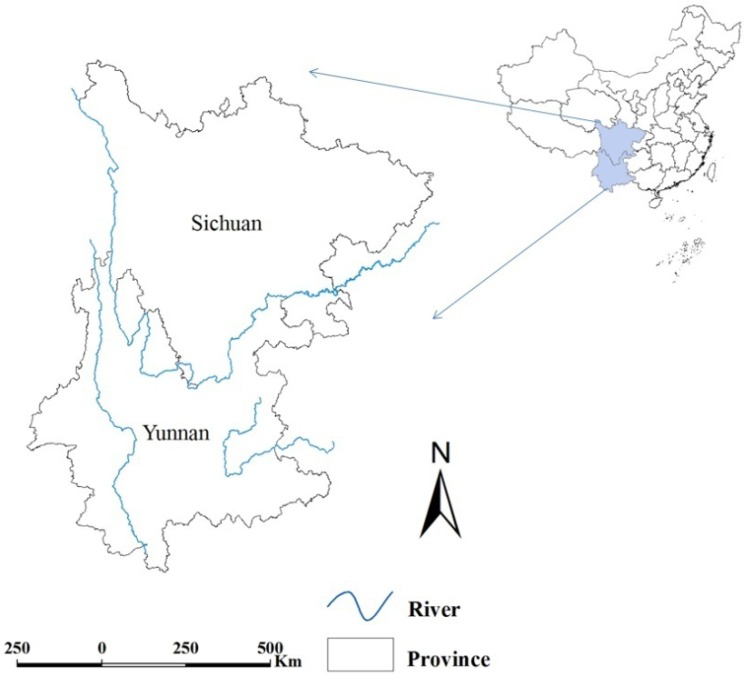
Location of Sichuan and Yunnan Province. Geographical rivers layer was overlaid. The map was created using the ArcGIS software (version 10.0, ESRI Inc., Redlands, CA, USA).

Techniques of geographic information system (GIS) and methods of cluster detection were combined to evaluate temporal changes in the spatial distribution of *S. japonicum*. The analysis was conducted at the county level within the two mountainous provinces of Southwest China, Sichuan and Yunnan where the WBLP was focused (Figure 1). Sichuan ranks as both the 3rd largest (485,000 square kilometers) and 4th most populous province (80.4 million in 2010) in China. Adjacent to Sichuan, Yunnan is a province spanning approximately 394,000 square kilometers and with a population of 45.9 million (2010). Most of the both provinces are mountainous or hilly, with plains and plateaus covering the rest of the land area.

### 2.2. Parasitological Data

National schistosomiasis datasets for the two analysis periods (1999–2001 and 2007–2008) were provided by Fudan University (formerly Shanghai Medical University) and the National Institute of Parasitic Diseases in Shanghai, Chinese Center for Disease Control and Prevention. The databases are county-based, with all reported schistosomiasis cases and the population at risk in each county included. Additional details describing the process of data collection can be found elsewhere [13].

### 2.3. Statistical Analysis

*Preliminary description of prevalence of schistosomiasis*. Mapping of prevalence of schistosomiasis for each year was performed to assess the spatial distribution of the epidemic over time. Spatial autocorrelation was investigated using the Global Moran’s *I* statistic [14].

*Detection of spatial clusters*. For each year, spatial clustering was assessed using Local Moran’s *I* [15] and Kulldorff statistic [16] to identify high-risk areas. These two methods provide complementary information on clustering patterns to identify areas of relatively high prevalence. The Local Moran’s *I* statistic provided a measure of the spatial autocorrelation for a given county with its neighbors. For both Global and Local Moran’s *I*, a pair of neighbor counties is defined with those sharing a common border. We assumed the weight element as 1 if two counties are neighbor and 0 otherwise in the weight matrix which defines the spatial relationships among counties. Spatial clusters of schistosomiasis cases are identified by finding local areas where high prevalence counties border other high prevalence counties (defined as high-high county) and where high counties border low prevalence counties (defined as high-low county). The Kulldorff’s statistic uses a moving window of pre-specified shape to identify single counties or groups of counties with statistically significant relatively high risk. We used an eclipse window in this study which moved throughout the study area.

*Frequency of occurrence of spatial clusters*. The frequency of spatial cluster occurrence was defined as the number of years during which the county contributed to a cluster and this was calculated for each county during the two study periods. Two types of frequencies were considered: (i) clusters detected by one of the two statistics above (weak evidence of clustering) and (ii) clusters detected by both statistics above (strong evidence of clustering). Mapping of prevalence of schistosomiasis was performed using ArcGIS software (version 10.0, ESRI Inc. Redlands, CA, USA) and the Global and Local Moran’s *I* statistic were calculated using the ArcGIS software as well. Spatial cluster analyses with Kulldorff’s statistic were implemented using the SaTScan software (version 8.0, Kulldorff and Information Management Services, Inc., Boston, MA, USA).

## 3. Results

**Figure 2:**
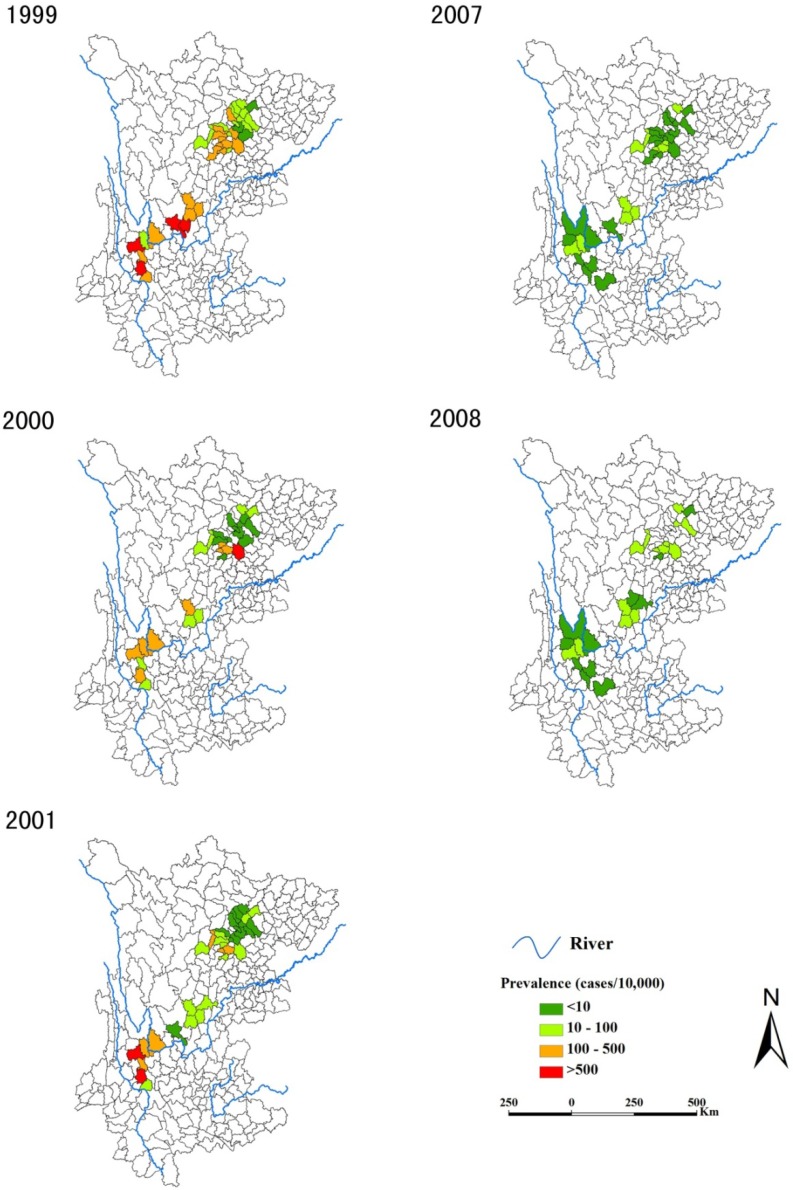
Annual prevalence of schistosomiasis at county level during the two study periods.

Figure 2 shows the annual prevalence of schistosomiasis during the two study periods, from which we can see that the burden of schistosomiasis decreased dramatically from the first (1999–2001) to the second (2007–2008) period. Actually, the mean prevalence of schistosomiasis decreased dramatically from 100.5 cases per 10,000 people in 1999 to 6.02/10,000 in 2008 during the two study periods (Table 1). This downward trend was accompanied by decreasing variation in prevalence across counties with the interquartile range (IQR) contracting from 0–162.2/10,000 in 1999 to 0–7.63/10,000 in 2008. The Global Moran’s *I* coefficient was particularly low with *p* > 0.05 for each year except 1999, indicating no significant overall spatial autocorrelation.

The maps were created using the ArcGIS software (version 10.0, ESRI Inc., Redlands, CA, USA).

**Table 1:** Prevalence (cases/10,000) of schistosomiasis and its global spatial autocorrelation in the mountainous area, China.

Year	Min	Q1	Mean	Q3	Max	*I*	*p*
1999	0	0	100.5	162.2	1172	0.13	0.04
2000	0	0	46.98	10.78	672	−0.03	0.55
2001	0	0	51.66	36.22	595.5	0.07	0.16
2007	0	0	6.85	5.69	70.73	0.11	0.07
2008	0	0	6.02	7.63	29.84	0.09	0.13

*I*: the Global Moran’s *I* coefficient; *p*: *p*-value for the Global Moran’s *I* statistic.

**Figure 3:**
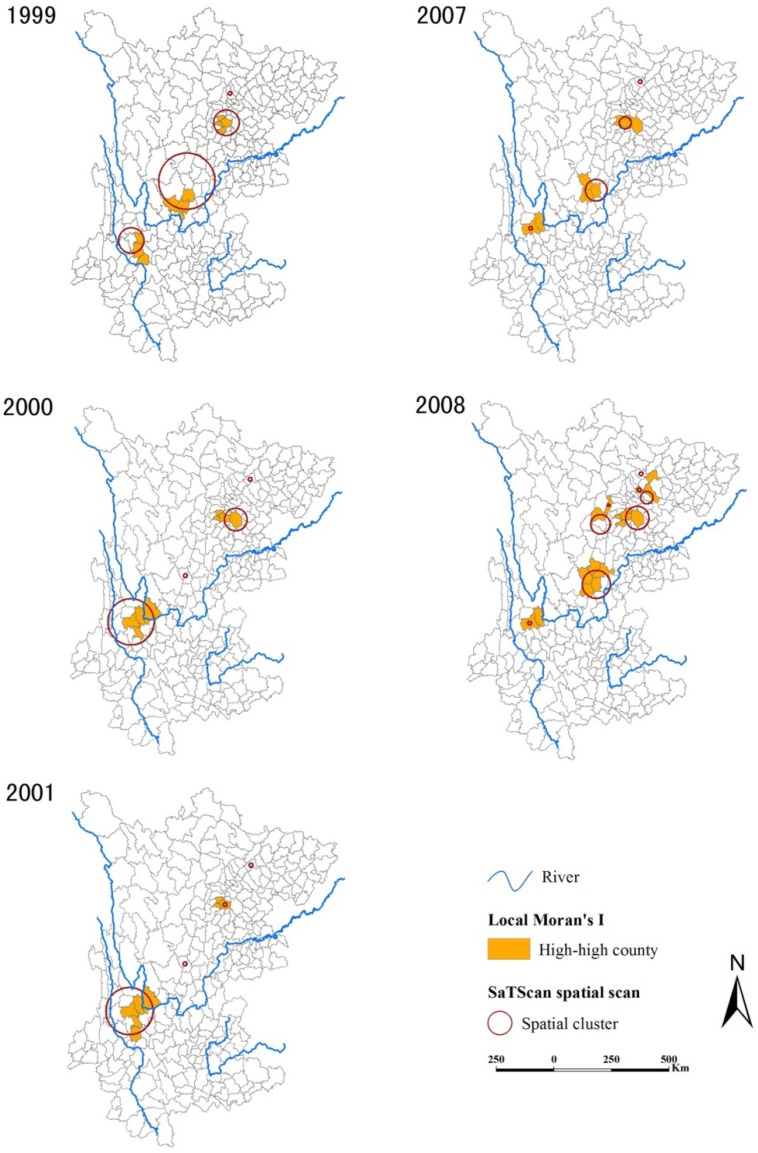
Annual spatial clusters of schistosomiasis during the two study periods.

Each panel shows for each year the results of both methods, the Anselin’s Local Moran’s *I* test and the Kulldorff’s spatial scan statistic. The maps were created using the ArcGIS software (version 10.0, ESRI Inc., Redlands, CA, USA).

Spatial clusters are shown in Figure 3, which were detected by both the Kulldorff’s spatial scan statistic and the Local Moran’s *I* results. The Local Moran’s *I* identified 14 high-high counties during 1999–2001, with an annual number ranging from 8 (in 2000 and 2001) to 10 (in 1999) and the median annual prevalence of counties flagged as being in high prevalence clusters of 216.79 (IQR = 160.03−450.81) cases per 10,000. In 2007–2008, the Local Moran’s *I* identified 18 high-high counties, with an annual number ranging from 10 (in 2007) to 17 (in 2008) and the median annual prevalence within these counties of 19.52 (IQR = 12.96−27.01) cases per 10,000. Note that no high-low counties were found during the two periods. The Kuldorff spatial scan method identified 12 different significant spatial clusters during 1999–2001 (four in each year); the median number of counties included in each cluster in this early period was 3 (IQR = 1−10). In 2007–2008, the Kuldorff spatial scan statistic identified 13 significant spatial clusters altogether (5 in 2007 and 8 in 2008); in this latter period the median number of counties contained within a cluster dropped to 1 (IQR = 1−3). Over the two study periods, almost 88% of the high-high counties identified with the Local Moran’s *I* were included in clusters detected by SaTScan and 64% of the SaTScan clusters encompassed high-high counties.

**Figure 4:**
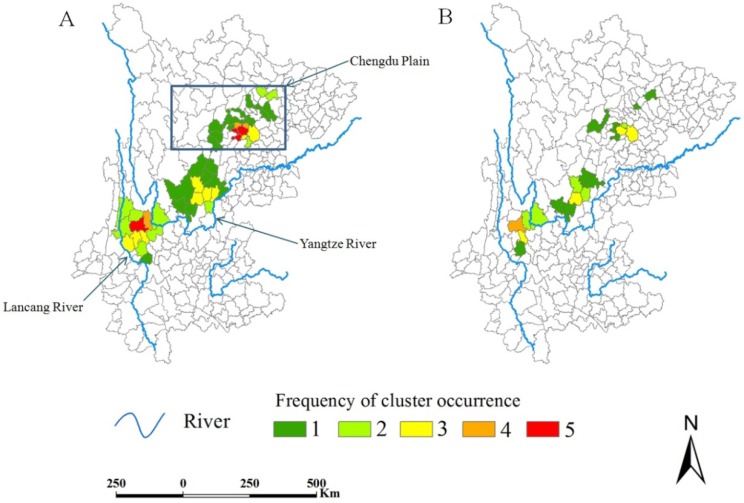
Frequency of cluster occurrence during the two study periods.

Spatial clusters generally occurred mainly in three regions (Chengdu Plain, Yangtze River Valley, and Lancang River Valley), but in different counties from year to year over the two study periods (Figure 4). Among the counties contributing to a cluster at least once, the median frequency was 2 (range = 1–5) for clusters detected by at least one method, and 1 (range = 1–4) for clusters detected by both methods. Five counties were detected three or more times by both methods (Renshou, Meishan, Dechang, Eryuan, and Dali).

(**A**) Frequencies of occurrence of spatial clusters detected by one of the two methods (Anselin’s Local Moran’s *I* test or Kulldorff’s spatial scan statistic); (**B**) Frequencies of occurrence of spatial clusters detected by both methods. The maps were created using the ArcGIS software (version 10.0, ESRI Inc., Redlands, CA, USA).

## 4. Discussion

This study presents an application of spatial cluster detection methods to examine changing patterns in the burden of schistosomiasis in hilly and mountainous areas in Southwest China during the period immediately following and 6–7 years after the end of the WBLP. Previous studies [8,17,18] have reported the evaluation of impact of the WBLP control strategies on schistosomiasis control, but most of these studies evaluated only the short-term impact of the WBLP, and did so from a non-spatial perspective [19]. Since different patterns of spatial distribution would require distinct control strategies, our study provides new insights and valuable information for future planning of schistosomiasis control efforts. To our knowledge, only Zhang *et al*. [13] have so far investigated spatial variations of schistosomiasis risk in this area using a Bayesian hierarchical model, but this analysis did not attempt to identify areas of spatial clustering. Spatial cluster analysis with time sequence provides unique insights since it provides valuable clues about potential disease sources (and determinants) [20], areas where clusters may recur [21], and promising areas to target for increased control efforts [22,23].

It was found that the overall prevalence of schistosomiasis had substantially reduced to a low level in the period of 2007–2008. While causality cannot be unambiguously attributed to the WBLP activities, we believe it is reasonable to conclude that these efforts may have been responsible for such impressive reductions in disease burden. Our analysis showed that there was a significant spatial autocorrelation of prevalence of schistosomiasis (*I* = 0.13, *p* = 0.04) in 1999 but no significant spatial autocorrelation existed in the following years, suggesting that the spatial distribution of the disease changed from a clustered pattern to a highly fragmented one.

Our spatial cluster analysis also demonstrated the greatly reduced burden of disease between these two periods: the median annual prevalence of counties within detected clusters decreased from 216.79 to 19.52 cases per 10,000 (Local Moran’s *I* results) and at a smaller scale (the median number of counties per cluster reduced from 3 to 1, Kulldorff’s spatial scan statistics). This reduction is evident in Figure 2. However, we found in the latter evaluation period that more clusters of high-risk counties were widely scattered than in 1999–2001 (Figure 3), indicating there were still large population at risk. This suggests that the WBLP strategy may have mitigated the burden of schistosomiasis but failed to effectively compress the spatial extent of the endemic areas.

It is well-known that the transmission of *S. japonicum* is closely related to the distribution of the snail intermediate host, which is associated with environmental conditions such as vegetation coverage, land-use patterns, quality and humidity of the soil [24]. In mountainous regions of Southwest China, snails are distributed in the paddy fields, irrigation ditches, and their adjacent meadows with water, where human and livestock (especially cattle) are frequently found [25]. The scattered clusters of schistosomiasis reflected the dispersed distribution of snail habitats to some extent. Possible causes of this pattern are environmental management [7], which included replacing farmland with forest or grass and turning rice paddies into cotton or corn land in recent years, and development of roads or highways with the social development in China [26], some of which passed through the snail habitats creating more snail habitats at a smaller scale.

In general, clusters occurred in different counties from year to year, but were more frequently found in five counties. Map A (Figure 4) shows that clusters mainly occurred in three regions: Chengdu Plain, Yangtze River Valley, and Lancang River Valley, indicating these regions should remain the focus for schistosomiasis control. Furthermore, the strong evidence of clustering, shown in Map B (Figure 4), implies that the five counties (four in yellow and one in orange) should be given priority in future schistosomiasis control efforts.

The control of *S. japonica* requires interruption of the parasite lifecycle by eliminating the intermediate host and the parasite from the definitive host and preventing infection of the intermediate or definitive host [27,28]. The provision of praziquantel in highly endemic regions is effective at reducing the infection rate [29,30], however, drug treatment alone may not be able to mitigate schistosome re-emergence or the establishment of new endemic areas [31]. This is exemplified by our study where, despite a decrease in prevalence, clusters became more scattered in 2007–2008. Therefore, an integrated control program [31] is required to combat schistosomiasis to achieve the aim of reducing human infection and prevalence to below 1% by 2015 in all endemic areas [32]. These interventions should include health education, access to clean water and adequate sanitation, mechanization of agriculture, fencing of water buffaloes, snail control, chemotherapy, and even future vaccination of livestock [6,33,34].

There are several limitations to the analysis presented here. First, our analysis depends on the reliability of reported surveillance data and stability in the efforts to detect cases over time. We do not believe that there have been substantial changes in reporting over the period of this analysis; Second, we are unable to unambiguously attribute changes in schistosomiasis prevalence to the WBLP since this was not a cluster randomized study and we do not have control areas with which to compare spatial patterns of disease burden over time; Third, methods of cluster detection we have used have limitations. For instance, the Local Moran’s *I* is biased when population at risk in each county varies substantially (on which prevalence is calculated) over the study area [35] and the Kulldorff’s spatial scan statistic requires that we pre-specify the shape of the clusters to be detected. We combined these two different approaches for cluster detection methods, as has been recommended by others [36], to help address these limitations and to improve our overall understanding of the complex and changing spatial distribution of schistosomiasis. Furthermore, the two methods show the trend of schistosomiasis from different aspects. The Local Moran’s *I*, based on spatial attribute (e.g., prevalence), reflects severity of schistosomiasis; while the kulldorff’s statistics, detecting spatial distribution, focuses on extent of schistosomiasis.

## 5. Conclusions

In summary, the period following the WBLP intervention was associated with substantial reductions in the burden of schistosomiasis, but limited impact on the spatial distribution of clustering within the study area. Clusters mainly occurred in three regions: Chengdu Plain, Yangtze River Valley, and Lancang River Valley, and that clusters were most frequently found in five counties, which must be targeted for increased interventions to speed the elimination of schistosomiasis.
